# Folate receptor alpha for cancer therapy: an antibody and antibody-drug conjugate target coming of age

**DOI:** 10.1080/19420862.2025.2470309

**Published:** 2025-03-05

**Authors:** Yi Liu, Xinyi Chen, Theodore Evan, Benjamina Esapa, Alicia Chenoweth, Anthony Cheung, Sophia N Karagiannis

**Affiliations:** a St. John’s Institute of Dermatology, School of Basic & Medical Biosciences & KHP Centre for Translational Medicine, King’s College London, Guy’s Hospital, London, UK; b Breast Cancer Now Research Unit, School of Cancer & Pharmaceutical Sciences, King’s College London, Guy’s Cancer Centre, Innovation Hub, Guy’s Hospital, London, UK

**Keywords:** ADC, antibody, folate receptor alpha, FRα, oncology, targeted therapy

## Abstract

Folate receptor alpha (FRα) has long been the focus of therapeutics development in oncology across several solid tumors, notably ovarian, lung, and subsets of breast cancers. Its multiple roles in cellular metabolism and carcinogenesis and tumor-specific overexpression relative to normal tissues render FRα an attractive target for biological therapies. Here we review the biological significance, expression distribution, and characteristics of FRα as a highly promising and now established therapy target. We discuss the ongoing development of FRα-targeting antibodies and antibody-drug conjugates (ADCs), the first of which has been approved for the treatment of ovarian cancer, providing the impetus for heightened research and therapy development. Novel insights into the tumor microenvironment, advances in antibody engineering to enhance immune-mediated effects, the emergence of ADCs, and several studies of anti-FRα agents combined with chemotherapy, targeted and immune therapy are offering new perspectives and treatment possibilities. Hence, we highlight key translational research and discuss several preclinical studies and clinical trials of interest, with an emphasis on agents and therapy combinations with potential to change future clinical practice.

## Introduction: overview of folate and folate transporters

Folate metabolism plays a key role in human biochemistry that becomes corrupted during tumorigenesis. It plays a central role in one-carbon metabolism, contributing to three fundamental biosynthetic pathways: de novo nucleoside synthesis, methylation reactions, and amino acid production.^1^ Due to its key contributions in carcinogenesis, folate metabolism has long been an attractive target in oncology drug development. The first antifolate drug, aminopterin, was among the earliest successful anticancer therapies, and its successor compounds, including methotrexate and pemetrexed, remain in widespread clinical use.^2^ More recent research has better characterized the mechanisms of folate transport in both normal and malignant tissues. Folate receptor alpha (FRα), a central mediator of folate uptake, has emerged as a uniquely attractive target for anticancer therapy. In this review, we emphasize the clinical potential of exploiting FRα in treatment of cancer, highlighting its growing relevance in advancing precision medicine.

Folate, also known as vitamin B9, is an essential nutrient for survival. Inadequate dietary intake can cause anemia and developmental defects.^3^ Dietary folate lacks biological activity and must be reduced to active tetrahydrofolate (THF) forms, such as 5-methyltetrahydrofolate (5-methyl-THF) and 5-formyltetrahydrofolate (5-formyl-THF).^4^ These THF derivatives carry different one-carbon units (i.e., methyl or formyl) which can be used for various cellular biosynthesis processes.^5^ A hydrophilic anionic molecule at physiological pH, folate does not readily diffuse across biological membranes. Uptake into mammalian cells relies on three major folate transporters: the reduced folate carrier (RFC), the proton-coupled folate transporter (PCFT), and folate receptors (FRs). These proteins are genetically distinct and functionally diverse, but all contribute to cellular folate balance. The most ubiquitously expressed folate transporter is RFC (SLC19A1), which bidirectionally transports the majority of dietary THF and clinically used antifolates at neutral pH, at high throughput and low affinity. The PCFT (SLC46A1) offers an alternative transmembrane route for folate uptake. In contrast with RFC, PCFT is a high-affinity folate-proton symporter under acidic conditions, whereby it mainly absorbs folates in the gastrointestinal tract. Finally, FRs are high-affinity low-throughput transporters that mediate unidirectional folate uptake through endocytosis, trafficking folate into highly acidic early endosomes.^6^

FRs, also known as folate binding proteins (FBPs), have four isoforms: FRα, FRβ, FRγ, and FRδ. These isoforms are encoded by the FOLR multigene family *FOLR1–4*. Although FRs share highly conserved consensus sequences in folate binding-related residues, exon variation leads to the diversity in folate binding affinities and tissue distribution.^7^ FRα is the most studied member of the family and is the central focus of this review, but the other FRs also play important roles in human physiology.

FRβ is present in the placenta, hematopoietic tissues such as the spleen, thymus, and bone marrow-derived myeloid cells.^8^ FRβ-expressing myeloid immune cells are present and vary dynamically during tumorigenesis, with the highest expression seen in tumor-associated macrophages (TAMs).^9^ FRβ is also considered a biomarker for M2 regulatory macrophage polarization and a potential emerging immunotherapy target.^10^ FRγ is unique among the FRs in that it is a secreted protein: it lacks the C-terminal hydrophobic glycophosphatidylinositol (GPI) membrane anchor that is found in all other FRs. It is mainly expressed in hematopoietic tissues,^8^ but its function remains poorly characterized. Quinn *et al*. identified FRγ as a stimulator of fibrogenesis in metabolic dysfunction-associated steatohepatitis by upregulated TGFβ activity in hepatic stellate cells.^11^ Recent studies have suggested FRγ and its single nucleotide polymorphism (SNP) variant could drive colony formation in chronic myeloid leukemia (CML) cells by promoting mitochondrial activity.^12^ Finally, in contrast to other isoforms, FRδ (JUNO) lacks the classic folate-binding pocket and exhibits very low affinity for folate.^13^ This isoform is expressed on the oocytes of several mammalian species, including humans, and binds specifically to the sperm cell-surface protein Izumo, playing a critical role in fertilization.^14^ FRδ may serve as a novel biomarker of CD4^+^ T follicular helper (T_FH_) cells during activation,^15^ although at present this function remains poorly understood.

## Folate receptor alpha in health and malignant disease

### Structure and normal tissue expression

**Figure 1.**
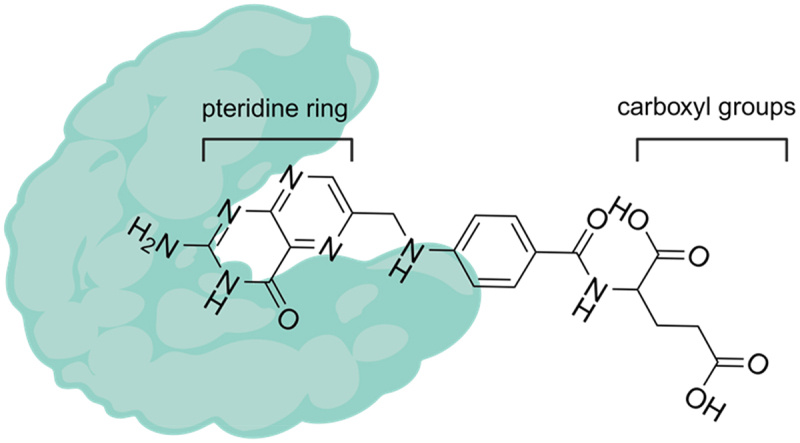
Schematic interaction between FRα and folic acid. Folic acid (chemical structure) protrudes from a deep binding pocket of the FRα (green cartoon model). The pteridine ring is buried in the pocket, while the two negative charged carboxyl groups of folic acid are placed outside the pocket, and this may allow folate-based conjugation.

FRα is a 38–40 kDa cysteine-rich glycoprotein with a carboxy-terminal GPI anchor. It has a globular structure that includes a deep open folate-binding pocket. The protein is stabilized by eight disulfide bridges formed by 16 conserved cysteine residues that link the core domains (six α-helices plus four β-strands). Upon binding, the basic pteroate moiety of folate is buried inside the pocket, while the two negatively charged carboxyl groups of folates extend past its positively charged entrance. These ‘stretching’ carboxyl groups of folate allow for folate-like molecules to be manipulated without adversely affecting FRα binding^7^ (Figure 1). Exploitation of this phenomenon has been central in the design of folate conjugate drugs and anti-FRα therapies.

In normal physiology, FRα is predominantly expressed in the apical or luminal surfaces of polarized epithelia that do not have direct access to circulating folate. Tissues known to express FRα include the choroid plexus, lung, thyroid, retina, and placenta.^16–18^ In addition, expression within the kidney is restricted to the proximal tubule, where FRα is thought to help reabsorb folate back into the circulation prior to urinary excretion.^19^

### Folate receptor alpha and its functions in health

#### FRα as a folate transporter

**Figure 2.**
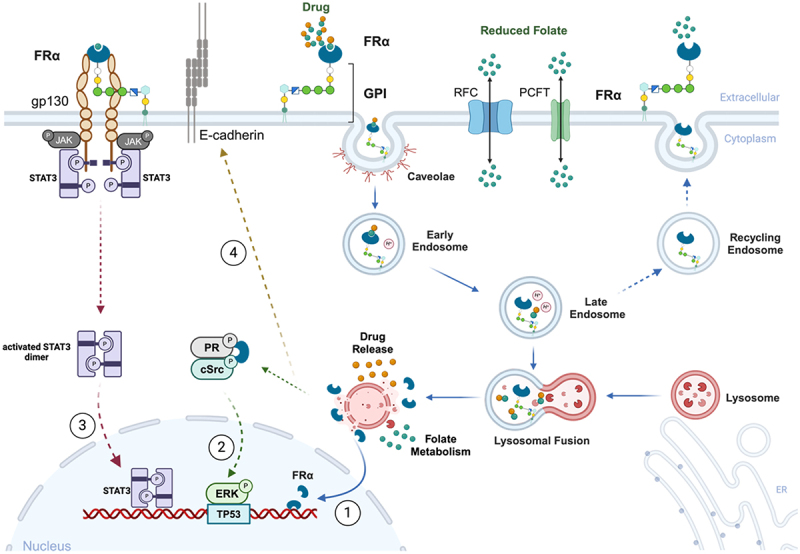
Proposed FRα-mediated endocytosis and signaling pathways in cells. GPI-anchored FRα is invaginated into caveolae vesicles and forms early-to-late endosomes, which undergo increasing acidification and subsequent fusion with lysosomes to release non-anchoring FRα and folate/drugs. Alternatively, the GPI-anchored FRα is recycled from late endosomes to cell membrane. Proposed signaling pathways associated with FRα are presented in ①-④. ①The GPI-specific phospholipase D cuts off the GPI anchor of FRα in lysosomes, resulting in the free FRα translocating into nucleus and acting as a transcription factor. ②FRα physically interacts with the Progesterone Receptor (PR) and the cellular proto-oncogene tyrosine-protein kinase (cSrc) in a trimeric complex. cSrc auto-phosphorylates to activate itself and promotes the phospho-activation of ERK. Activated ERK induces the transcription of TP53 to regulate cell cycle progression. ③Folate binding to FRα can induce the activation of the JAK/STAT3 signaling pathway via the gp130 co-receptor, leading to cell proliferation. ④FRα may upregulate the expression of cell-cell adhesion molecule E-cadherin to promote cell migration. Created with BioRender.com. Abbreviations: FRα, folate receptor alpha; GPI, glycosylphosphatidylinositol; RFC, reduced folate carrier; PCFT, proton-coupled folate transporter; PR, progesterone receptor; JAK, Janus kinase; cSrc, cellular proto-oncogene tyrosine-protein kinase; STAT3, signal transducer and activator of transcription 3.

The most well-characterized role of FRα is its function as a transporter. This process begins when folate derivatives, such as 5-methyl-THF, bind to GPI-anchored FRα in the cell membrane. Following ligand binding, receptor-ligand complexes cluster and invaginate into intracellular vesicles via endocytosis. These cytoplasmic vesicles are subsequently acidified and fused with lysosomes, where dissociation of the folates from FRα is thought to occur in order to facilitate their use in various metabolic processes^20^ (Figure 2). A more complex variant of this model has been described by Grapp and colleagues in the central nervous system. According to their work, after endocytosis, the receptor-ligand complexes are translocated into GPI-anchored early endosomal compartments (GEECs) and then transferred to multi-vesicular bodies (MVBs). Intraluminal vesicles from the membrane of MVBs bud inward and release directly into cerebrospinal fluid as exosomes, permeating through the ependymal layer and facilitating uptake by neurons.^21^

#### FRα as a transcription factor

It has been suggested that FRα can translocate to the nucleus and act as a transcription factor.^22^ Following the release of folate from lysosomes, the GPI-specific phospholipase D cleaves the GPI anchor of FRα in lysosomes, resulting in free FRα translocating into the nucleus^22^ (Figure 2). Although a detailed characterization of the mechanism remains elusive, published data suggest that nuclear FRα can bind to *cis*-regulatory elements and directly regulate transcription, although how this process is regulated in health and disease remains unknown.^23^

#### FRα in embryogenesis

Accumulating evidence suggests that FRα plays a critical role in embryogenesis. Mice lacking FRα have severe birth defects, including neurological and cardiovascular anomalies,^24^ and die during development. This phenotype can be reversed through supplementation with folic acid.^25^ FRα contributes to neural tube formation in embryos,^26^ with particularly high expression in the neural folds and in the yolk sac, suggesting it may mediate maternal-fetal folate transport during neurulation.^27^ Dysfunctional FRα is also associated with a neurological syndrome in newborns.^28^ Together, these findings underpin a central role in neural development.

### Folate receptor alpha functions in tumorigenesis

**Figure 3.**
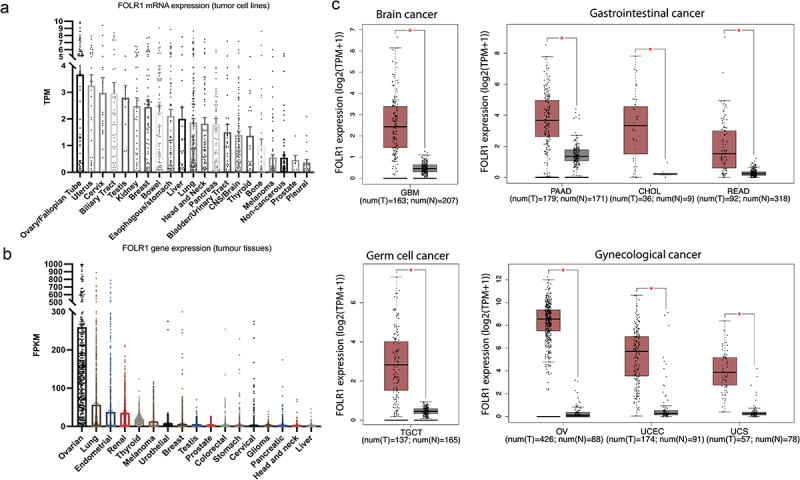
FOLR1 expression in malignant and normal tissues. a, FOLR1 mRNA expression, derived from RNA-seq data, across cell lines of different solid tumor types (data from DepMap Public 24Q4, OmicsExpressionProteinCodingGenesTPMLogp1.csv, https://depmap.org/portal). TPM = transcripts per million. b, FOLR1 gene expression in human tissues across cancer types (from TCGA data of the Human Protein Atlas,https://v20.proteinatlas.org). FPKM = number of fragments per kilobase of exon per million reads. c, Comparison of FOLR1 gene expression between tumor and adjacent normal tissues (from GEPIA, http://gepia.cancer-pku.cn). GBM, Glioblastoma multiforme; PAAD, Pancreatic adenocarcinoma; CHOL, Cholangial carcinoma; READ, Rectum adenocarcinoma; TGCT, Testicular Germ Cell Tumors; OV, Ovarian serous cystadenocarcinoma; UCEC, Uterine Corpus Endometrial Carcinoma; UCS, Uterine Carcinosarcoma. Student’s t test: *p ≤ 0.05.

Akin to many genes important in human development, FRα physiology is also disrupted in cancer. Elevated FRα expression is found in a large number of human carcinomas, including ovary, endometrium, lung, brain and gastrointestinal malignancies^18^ (Figure 3). Nevertheless, the function of FRα in tumors remains insufficiently characterized, though several potential tumorigenic roles have been described.

In the first instance, FRα, as an important folate transporter, appears to promote tumorigenesis by supplying folate to fuel cancer cell metabolism. Bistulfi et al. showed that mild folate depletion induces genetic and epigenetic instability in prostate cancer cells compared with those supplemented with higher levels of folate.^29^ However, as folate homeostasis is predominantly controlled by RFC, the importance of FRα in mediating this phenotype remains controversial. A meta-analysis suggested dietary folate intake may reduce the risk of developing breast cancer risk, complicating this relationship further.^30^

Alternatively, it has been proposed that the contributions of FRα in cancer development are primarily driven by its transcription factor function. Nuclear translocation of FRα is associated with increased proliferation and reprogramming toward a ‘cancer stem cell’ through activation of numerous transcription factors, including OCT4/SOX2 (regulating stemness), KLF4 (regulating cell reprogramming), HES1 (regulating metastasis and multidrug resistance), and FGFR4 (regulating cellular proliferation).^22,23^

Finally, emerging evidence suggests that FRα directly interacts with signaling pathways that promote tumorigenesis (Figure 2). For example, folate-FRα complexes can activate oncogenic STAT3 in a JAK-dependent manner with the aid of the co-receptor gp130.^31^ FRα is also thought to interact with the serine/threonine kinases ERK1/2, core mediators of the mitogen-activated protein signaling pathway. This pathway was proposed by Lee and colleagues, who described a direct interaction between FRα and cSrc (cellular proto-oncogene tyrosine-protein kinase) with the aid of the progesterone receptor in colon cancer cell lines. This Trimeric complex promotes the activation of ERK signaling pathway and thus may regulate TP53 gene transcription.^32^ In addition, in ovarian cancer, knockdown of FRα abrogates cellular proliferation and migration, potentially through downregulation of the cell–cell adhesion molecule E-cadherin.^33^ These data suggest that the oncogenic functions of FRα extend well beyond its canonical role in metabolism.

## Folate receptor alpha as a target for antibody-based therapeutics

FRα is increasingly viewed as an attractive anticancer target for several reasons. Firstly, it is highly expressed in malignant tissues relative to normal tissue counterparts (Figure 3), and FRα expression is no longer polarized to a particular tissue area.^34^ Secondly, higher levels of FRα expression correlate with disease stage as well as recurrence in a variety of solid tumors, including ovarian cancer, triple negative breast cancer, and lung cancer.^35–37^ This highlights the potential prognostic role for FRα and further emphasizes its promise as a target in advanced disease. The ‘built-in’ endocytosis-dependent absorption pathway denotes the potential for intracellular drug delivery in the context of an ADC.^38^ Finally, the relatively minor role of FRα in physiological folate uptake (compared with RFC) in nonmalignant adult tissues, compared with its enhanced folate uptake in cancer, may offer increased potential therapeutic index as a drug target.

### Monoclonal antibodies

#### Farletuzumab

Farletuzumab (MORAb-003) is a humanized IgG1 monoclonal antibody recognizing FRα. It was modified from a murine LK26 clone identified by immunization,^39^ then selected and optimized to maintain a similar antigen binding affinity to the original murine form (K_D_ = 2 µM).^39^ Despite high affinity, farletuzumab does not interfere with folate binding and uptake into cells, nor does it block the accumulation or activity of anti-folate compounds.^40^ Instead, farletuzumab promotes cell death through Fc-mediated mechanisms, including antibody-dependent cellular cytotoxicity (ADCC) and complement-dependent cytotoxicity (CDC).^39^ Furthermore, sustained binding to FRα induces autophagy and suppresses cell proliferation.^41,42^

Farletuzumab was first developed as a potential monotherapy in Phase 1 clinical trials of patients with FRα-expressing solid tumors. No dose-limiting toxicities (DLTs) were encountered,^43^ and adverse effects were generally mild.^44^ In a subsequent Phase 2 trial (NCT00318370) (Table 1), farletuzumab was given to patients with platinum-sensitive recurrent ovarian cancer weekly, as a single agent or alongside carboplatin and taxane chemotherapy every 21 days for six cycles.,^46^ Of 47 subjects who received farletuzumab with carboplatin and taxane, the combination therapy resulted in a 75% complete or partial response rate and 21% of patients with stable disease. No additional toxicity was observed when combining farletuzumab with the standard carboplatin/taxane chemotherapy. In addition, more than 20% of patients experienced a longer progression-free period than their first response period after their first treatment. Collectively, the findings of high response rate, longer response duration, and the absence of additional toxicity, suggested that the addition of farletuzumab to chemotherapy may be beneficial to patients with recurrent ovarian cancer. These early data culminated in one of the first Phase 3 studies of a FRα-targeting therapy, platinum-resistant ovarian cancer. Farletuzumab given as carboplatin/taxane combination therapy was continued in a Phase 3 clinical trial (NCT00849667) (Table 1). In this later Phase 3 clinical trial, a total of 1,100 women with platinum-sensitive relapse were given carboplatin/taxane and either farletuzumab (1.25 mg/kg or 2.5 mg/kg) or placebo.^46^ The additional farletuzumab did not introduce new safety issues in patients. However, neither farletuzumab dose significantly enhanced progression-free survival compared to the placebo group.

**Table 1.** Examples of antibodies and T cell therapies in clinical development targeting FRα.

Antibody Name	Developer	Clinical Trial Number (Phase and number of patients)	Tumor Type	Antibody Isotype / Composition	Status/Outcome	Ref
**Antibodies**						
Farletuzumab (MORAb-003)	Morphotek	1. MORAb-003-002; NCT00318370 (Phase II, 58 patients)	Relapsed ovarian cancer after platinum-based chemotherapy	Humanized IgG1	Completed. Enhanced response rate and duration of response.	[43–47, 54, 55]
	Morphotek	2. MORAb-003-003; NCT00738699 (Phase III, 417 patients)	Platinum-resistant or refractory relapsed ovarian cancer	Humanized IgG1	Humanized IgG1 Terminated. Pre-specified criteria for continuation not met following futility analysis.	
	Morphotek	3. MORAb-003-004; NCT00849667 (Phase III, 1100 patients)	Platinum-sensitive ovarian cancer in first relapse	Humanized IgG1	Terminated. Lack of efficacy.	
	Morphotek	4. MORAb-003-005; NCT01004380 (Phase I, 15 patients)	Platinum-sensitive ovarian cancer	Humanized IgG1	Completed. Safety profile is tolerable. Evidence of anti-tumor activity.	
	Eisai Inc	5. MORAb-003-011; NCT02289950 (Phase II, 332 patients)	Low CA125 Platinum-sensitive ovarian cancer	Humanized IgG1	Completed. Improved PFS not met.	
	Morphotek	6. MORab-003-009; NCT01218516 (Phase II, 130 patients)	Stage IV adenocarcinoma of the lung	Humanized IgG1	Completed. Data awaited	
MOv18 IgE	King’s College London & Cancer Research UK	1. NCT02546921 (Phase Ia, 26 patients)	FRα-expressing ovarian cancer and other gynecological cancers	Mouse / human Chimeric IgE	Completed. Safety profile is tolerable. Evidence of anti-tumor activity.	[48]
	Epsilogen Ltd	2. NCT06547840 (Phase Ib, will enroll 45 patients)	FRα-expressing platinum-resistant ovarian cancer (PROC) whose disease has progressed after no more than four lines of prior therapy	Mouse / human Chimeric IgE	Open, recruiting. Will assess the safety, tolerability and efficacy of MOv18 IgE in ascending dose cohorts.	
**T Cell Therapies**						
MOv19-BBz CAR T Cells	University of Pennsylvania	NCT03585764 (Phase I, 46 patients)	FRα-expressing recurrent high grade serous ovarian, fallopian tube or primary peritoneal cancers	T cells engineered with Chimeric MOv19 scFv plus CD3ζ and 4-1BB (CD137) signaling domains	Terminated. Halted prematurely due to recruitment barriers.	[49]
ITIL-306	Instil Bio	NCT05397093 (Phase Ia/Ib, will enroll 51 patients)	Epithelial ovarian cancer, non-small cell lung cancer and renal cell carcinoma	Tumor-infiltrating lymphocytes engineered with costimulatory anti-FRα antigen receptor	Active, not recruiting	[50]

Nevertheless, subgroup analysis suggested that patients with lower CA-125 level had enhanced response and longer survival in the higher farletuzumab dose group, suggesting that patients with lower CA-125 levels may demonstrate an enhanced immune response mediated by farletuzumab, thus improved therapeutic outcomes when adding farletuzumab to chemotherapy. Based on these findings, a follow-on clinical trial investigated this farletuzumab regimen in patients with platinum-sensitive ovarian cancer who have lower CA-125 level at baseline. In a later Phase 2 trial (NCT02289950) (Table 1), patients with recurrent ovarian cancer and lower CA-125 level were given chemotherapy and either farletuzumab or placebo.^47^ However, the addition of farletuzumab to standard chemotherapy did not significantly improve efficacy compared to placebo. Interestingly, unlike the previous trials, most patients were treated with carboplatin and pegylated liposomal doxorubicin (PLD) instead of taxane. In the absence of farletuzumab, the overall response rate and progression-free survival were improved in the carboplatin/PLD group compared to the carboplatin/taxane group. This superior therapeutic outcome was also seen in other studies.^51–53^ Due to the unexpectedly high response rate in the placebo group, potentially because of the increased PLD efficacy, it might be more difficult to demonstrate additional benefits with farletuzumab. Nevertheless, no additional toxicity from farletuzumab was seen in this trial and an early Phase 1b clinical trial (NCT01004380)^47,54^ (Table 1). Similar disappointing results were reported from a trial of farletuzumab and paclitaxel combination in another platinum-resistant ovarian patient cohort, resulting in the termination of the trial following interim results analysis (NCT00738699) (Table 1).

One study also evaluated the effect of farletuzumab labeled with the alpha-emitter astatine-211 in mice with subcutaneous ovarian tumors.^55^ Remarkably, the ^211^At-labeled farletuzumab demonstrated a significant improvement in antitumor activity compared to farletuzumab or a nonspecific ^211^At-labeled antibody. These findings await translation into early-phase clinical studies.

#### KHK2805, a novel Fc-enhanced antibody

KHK2805, a novel humanized antibody, was generated from a rat clone, with high affinity to a different epitope of FRα. This antibody was designed in a defucosylated form by production in a FUT8-knockout expression host, resulting in enhanced ADCC and CDC activity. This antibody showed remarkable activity in ovarian cancer cell lines and patient-derived models as monotherapy, in metastatic, platinum-resistant preclinical models of ovarian cancer,^56^ but no further development has been documented.

#### MOv19

The murine IgG2a antibody MOv19 was generated by hybridoma technology in the late 1980s, using the crude membrane of an ovarian cancer cell line as immunogen.^57^ Both MOv19 and LK26, the original mouse clone of falertuzumab, recognized overlapping FRα epitopes with high affinity.^58^ The murine constant regions of MOv19 (γ2a, κ) were replaced with human CL (κ) and CH (γ1) to generate the chimeric antibody ChiMOv19, which exhibited similar or superior ADCC activity than its murine counterpart.^59^ Subsequently, a humanized derivative of MOv19 (denoted M9346A) was generated. This construct now serves as the antibody moiety of the ADC mirvetuximab soravtansine (IMGN853).^60^

MOv19 has been widely engineered into various fragments. For instance, murine MOv19 as a single-chain Fv (scFv) format has been fused with IL-2 to improve tissue penetration of IL-2,^61^ and with the retention signal KDEL designed to block the expression of FRα on the surface of cancer cells.^58^ The human Fab fragment AFRA5 was optimized into a chemical dimer AFRA 5.3 DFM.^62^ In preclinical intraperitoneal murine models, when radio-labeled with ^131^I, this Fab dimer specifically bound to FRα-expressing ovarian cancer cells, efficiently localizing to tumor masses due to its small size, where it promoted tumor regression and improved survival.^63^

MOv19 was also the first antitumor antibody used for generating bispecific antibodies (BsAbs) in solid tumors. In the early 1990s, a CD16/MOv19 BsAb was generated to trigger the specific lysis of ovarian cancer cells by natural killer cells. Similarly, a T cell BsAb, combining the FRα specific binding moiety of MOv19 with a monovalent anti-human CD3 antibody, was shown to activate intratumoral T cells and induce tumor cell death; its efficacy was shown to be dependent on the presence of functional T cells in the microenvironment.^64^

#### MOv18 IgG1

MOv18 was generated in parallel to MOv19 from a separate hybridoma clone, by immunizing mice with human ovarian cancer tissue.^59^ The mouse MOv18 clone was radiolabeled with ^131^I and administered to patients intravenously or intraperitoneally, to assess the clinical feasibility of radio-immuno-scintigraphy (RIS). Although tumor uptake and was observed with limited toxicities reported, nearly all patients developed human anti-mouse antibodies (HAMA).^65^

To mitigate the HAMA responses, the murine CL (κ) and CH (γ1) regions were replaced with their human equivalents (γ1, κ) to generate the chimeric MOv18 antibody, which exerted ADCC activity against tumor cells.^59^ Radiolabeled chimeric MOv18 IgG1 was evaluated in several early phase trials, where it was found to localize effectively to ovarian cancer tissue. Treatment was associated with limited side effects (e.g., fever, headache, and nausea). No human anti-chimeric antibody (HACA) responses were detected up to 12 weeks post-injection,^66^ but no further development has been documented.

#### MOv18 IgE

MOv18 IgE is a chimeric first-in-class IgE antibody specific for FRα, composed of the murine V regions of the MOv18 clone and human Cε regions.^67^ The use of IgE Fc regions in antibodies for cancer immunotherapy of solid tumors is based on several advantageous attributes of IgE which are different to IgG1. These include very high affinity for cognate FcɛRs expressed on immune effector cells including those found to infiltrate tumor lesions, and the absence of inhibitory Fc receptors. These features harbor the potential to result in long tissue residency, enhanced potency, and longevity of cancer-specific immune responses.^68^

In preclinical models, MOv18 IgE-induced cancer cell death by macrophage/monocyte dependent cytotoxic and phagocytic (ADCC and ADCP) mechanisms, as well as by promoting secretion of pro-inflammatory and macrophage chemotactic mediators such as tumor necrosis factor and monocyte chemoattractant protein-1.^69,70^ MOv18 IgE also demonstrated superior efficacy compared to IgG1 in patient-derived tumor xenograft models in mice^67,71,72^ and in immunocompetent rat models of cancer.^69^ One of the key concerns for the use of IgE class therapeutics in patients with cancer is the potential for the IgE therapeutic candidate to bind on the surface of basophils and be crosslinked by circulating multivalent antigen or autoantibodies. This may lead to basophil degranulation and type I hypersensitivity. MOv18 IgE was the first IgE therapeutic to be tested both preclinically and in a Phase 1 clinical trial. Pre-clinically, two in vitro/ex vivo tests provided early evidence of safe administration of MOv18 IgE in patients with cancer: a mast cell degranulation assay conducted in the presence of serum from ovarian cancer patients, and the Basophil Activation Test (BAT), conducted in whole unfractionated human blood, an emerging clinical tool used in allergy, to evaluate propensity for type I hypersensitivity to different allergens oncology therapeutic agents such as chemotherapies and therapeutic antibodies. Both the mast cell degranulation assay and the BAT showed that MOv18 IgE, when incubated in the presence of ovarian cancer patient sera and whole blood, respectively, showed lack of mast cell and basophil stimulation, suggesting the absence of type I hypersensitivity, and thus low risk for systemic anaphylaxis.^73^

Recently, a Phase 1 clinical trial of MOv18 IgE demonstrated a very good safety profile in patients with tumors expressing FRα and provided early evidence of clinical response.^48^ A Phase 1b study in platinum-resistant ovarian cancer is currently recruiting (Table 1).

#### T cell therapies directed against FRα

Chimeric antigen receptor (CAR) T cells have introduced a new era for cancer immunotherapy. By engineering an antigen-specific single-chain variable-fragment antibody (scFv) fused to intracellular lymphocyte signaling domains, T cells are functionally redirected to specific surface molecules on tumor cells.^74^ Although CAR T cell therapy for non-hematopoietic solid tumors remains challenging compared to application in B-lineage malignancies, ongoing research and clinical trials include anti-FRα CAR T cells.

A therapeutic strategy on FRα-expressing tumors was designed by engineering autologous T cells with the scFv of the murine MOv18 clone and a signaling domain of the Fc receptor γ chain to treat metastatic ovarian cancer. The Phase 1 trial demonstrated the safe administration of gene-modified T cells to patients with FRα expressing tumors.^75^ For enhancing T cell activation and proliferation, MOv19-BBζ, a second-generation anti-FRα CAR T cell construct with combined intracellular CD3ζ and 4-1BB (CD137) costimulatory signaling domains, was developed by Powell and colleagues and tested *in vivo*. MOv19-BBζ showed improved T cell persistence and potent antitumor activity.^76^ The first-in-human Phase 1 clinical trial was launched to evaluate the safety of MOv19-BBζ CAR T cells in patients with FRα-expressing recurrent high grade serous ovarian cancer (NCT03585764).^49,77^ Pilot studies of anti-FRα CAR constructs are being conducted against gastric cancer and triple-negative breast cancer.^78,79^

Despite the significant promise of CAR T cell therapy, several limitations overcome to achieve successful translation to the clinic. It is possible that murine-derived scFv sequences may trigger HAMA responses, leading to impaired anti-tumor efficacy and immune-related toxicity,^80^ and fully human CAR candidates may be required. The murine MOv19 scFv was successfully converted to the humanized anti-FRα scFv C4 construct for the purpose of reducing immunogenicity in humans. This FRα-targeting domain was coupled to CD3ζ and CD27 signaling domains in tandem to generate an anti-FRα CAR T cell therapy. This construct showed comparable anti-tumor cytotoxic activity to the murine counterpart *in vitro* and *in vivo*, and reduced risk of transgene immunogenicity and on-target/off-tumor toxicity.^81^ CARs stimulated ex vivo prior to administration and administered in combination with different cytokines have been reported in preclinical studies.^80,82^

Tumor-infiltrating lymphocyte (TIL) therapy is designed to manipulate T cells collected from patient tumors. TILs in combination with FRα targeting molecules have been designed and studied. For example, ITIL-306 is an autologous TIL therapy that combines T cell receptor-specific antigen recognition (against the HLA-A *02/MART-1 antigen) with the costimulatory signal of anti-FRα, designed to increase TIL activity in the presence of FRα-expressing tumor cells. This therapy showed sustained T cell proliferation and enhanced antitumor activity in the absence of exogenous IL-2 stimulation.^83^ Based on promising preclinical results, a multicancer Phase 1 dose escalation and expansion study evaluating the safety and feasibility of ITIL-306 in adult patients with advanced solid malignancies is active and recruiting (NCT05397093).^50^

### Antibody-drug conjugates targeting FRα-expressing tumors

Antibody-drug conjugates (ADCs) are therapeutics consisting of an antibody and cytotoxic drug payloads with inherent antitumor activity, joined by a chemical linker. An ADC combines the specificity of an antibody with the toxicity of payloads to selectively target and kill target antigen-expressing malignant cells, while in principle sparing healthy cells. This allows for the administration of powerful drugs that would ordinarily be too toxic to be delivered alone.

#### Mirvetuximab Soravtansine

**Figure 4.**
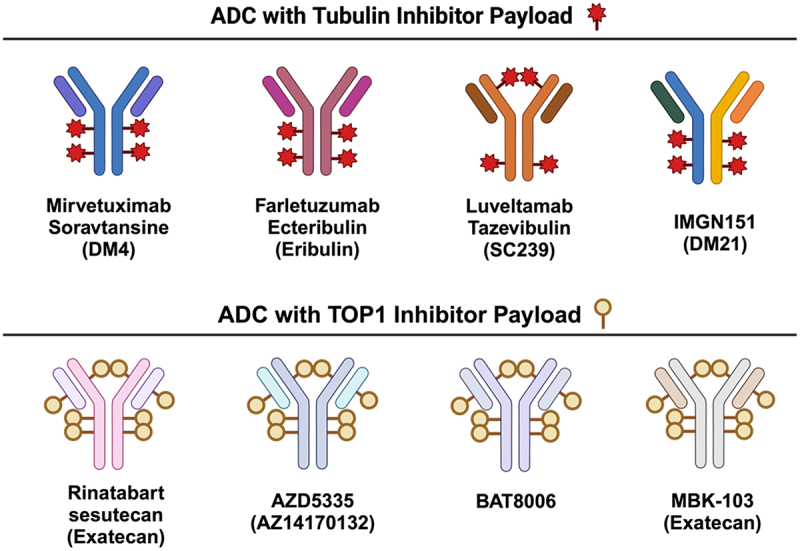
Schematic diagrams of antibody-drug conjugates (ADCs) against FRα in clinical trials. Approximate drug-antibody ratios (DAR) for each ADC are presented. ADC payloads that function by inhibition of tubulins are shown on the top panel, and those carrying topoisomerase 1 (TOP1) inhibitors are shown on the bottom panel. Payloads are stated in brackets. Created with BioRender.com.

Mirvetuximab soravtansine-gynx (MIRV, IMGN853, Elahere), developed by ImmunoGen, is the first licensed ADC targeting FRα. It is formed by a derivative of the MOv19 clone, the humanized antibody M9346A, joined to the cytotoxic maytansinoid DM4 by a cleavable disulfide linker (sulfo-SPBD)^60^ (Figure 4). Upon cellular uptake, MIRV is internalized via FRα-mediated endocytosis, trafficked to lysosomes and reduced by glutathione to release DM4 and S-methyl-DM4. These metabolites arrest cell cycle in prometaphase/metaphase, by suppressing microtubule stability during mitosis and thus inhibit the formation of the mitotic spindle, to potentiate cell death.^84^ The cytotoxic payloads also diffuse into the surrounding intercellular space, killing FRα-negative cells through bystander killing.^60^ In preclinical studies, MIRV displayed cytotoxic activity in a wide variety of FRα-expressing tumor models.^60^

This promising preclinical data propelled MIRV through rapid clinical development. Results from a pivotal Phase 1 expansion study (NCT01609556) reported in 2017 demonstrated the first evidence of efficacy in platinum-resistant ovarian cancer (PROC), with an objective response rate (ORR) of 26% and progression-free survival (PFS) of 4.8 months in a heavily pretreated patient cohort.^85^ The subsequent *FORWARD I* Phase 3 trial (NCT02631876) failed to demonstrate improved PFS for MIRV compared to chemotherapy in FRα-positive PROC patients who received 1–3 prior therapies (Table 2). In *FORWARD I*, patients were classified as FRα positive by an immunohistochemical assay. A later analysis suggested the criteria used in the trial were too permissive, and that responses were observed in a high FRα-expressing subpopulation.^86^ Building on this, the *SORAYA* (Phase 2) and *MIRASOL* (Phase 3) trials were designed to better evaluate MIRV in a ‘true’ FRα-high PROC population. The *MIRASOL* trial (NCT04209855) in particular firmly demonstrated the efficacy of MIRV versus chemotherapy in patients with high-grade serous PROC, with an ORR of 42.3% and overall survival of 16 months.^98^ As a result, MIRV received accelerated approval from the US Food and Drug Administration in 2022 for the treatment of adults with FRα-positive ovarian, fallopian tube, or primary peritoneal, cancers, after failure of at least one standard treatment regimen^99^ (Table 2).

**Table 2.** Examples of ADCs in clinical development targeting FRα.

ADC Name	Developer	Clinical Trial Number (Phase and number of patients)	Tumor Type	Payload	Payload Action	Linker	DAR	Status/ Outcome	Ref
Mirvetuximab Soravtansine-gynx (IMGN853/ Elahere ®)	Immuno-Gen	1. FORWARD I; NCT02631876 (Phase III, 366 patients)	Platinum-resistant ovarian cancer	DM4 (maytansine)	Tubulin inhibitor	Sulfo-SPDB	3.5 to 5	FDA approved, 2022	[85–88, 98–100, 107–109]
	AbbVie	2. MIRASOL; NCT04209855 (Phase III, 453 patients)	Platinum-resistant ovarian cancer					Completed, Benefit over chemotherapy	
	AbbVie	3. GLORIOSA, NCT05445778 (Phase III)	Platinum-sensitive ovarian Cancer					On-going	
	Immuno-Gen	4. SORAYA; NCT04296890 (Phase III, 106 patients)	Platinum-resistant ovarian cancer					Completed, Antitumor activity. Well-tolerated.	
	AbbVie	5. PICCOLO; NCT05041257 (Phase II, 79 patients)	Platinum-sensitive ovarian Cancer					Completed. Data awaited	
	Alessandro Santin & Yale University	6. NCT03832361 (Phase II, 50 patients)	Endometrial cancer					On-going	
	ImmunoGen, Inc.	7. NCT01609556 (Phase I, 206 patients)	Ovarian cancer and solid tumors					Completed. Manageable safety profile.	
	AbbVie	8. NCT06682988 (Phase III, 110 patients)	Platinum-Resistant Advanced High-Grade Epithelial Ovarian, Primary Peritoneal, or Fallopian Tube Cancers with High Folate Receptor-Alpha Expression & Platinum-resistant ovarian cancer with moderate hepatic impairment					On-going	
	M.D. Anderson Cancer Center	9. NCT03106077 (Phase II, 96 patients)	Triple-negative breast cancer					Completed. Data awaited	
Farletuzumab Ecteribulin (MORAb-202)	Eisai Inc	1. NCT03386942 (Phase I, 22 patients)	FRα-positive solid tumors	Eribulin	Tubulin inhibitor	Valine-citrulline	4	Completed. Preliminary antitumor activity. Well-tolerated.	[89, 90, 101]
	Bristol-Myers Squibb	2. NCT05613088 (Phase II, 90 patients)	Platinum-resistant ovarian cancer					On-going	
	Bristol-Myers Squibb	3. NCT05577715 (Phase II, 50 patients)	Non-Small Cell Lung Cancer (NSCLC) Adenocarcinoma (AC)					Terminated. Business objectives have changed.	
	Eisai Inc	4. NCT04300556 (Phase I/II, 142 patients)	Ovarian cancer, endometrial cancer, non-small cell lung carcinoma and triple-negative breast cancer					On-going	
AZD5335	Astrazeneca	FONTANA; NCT05797168 (Phase I/IIa, 396 patients)	Ovarian cancer	AZ14170132	TOP1 inhibitor	Valine-alanine	8	On-going	[91]
Rinatabart sesutecan (PRO1184)	Genmab	1. NCT05579366 (Phase I/II, 404 patients)	Advanced solid tumors	Exatecan	TOP1 inhibitor	NA	8	On-going	[92]
	Genmab	2. NCT06619236 (Phase III, 530 patients)	Platinum-resistant ovarian cancer					On-going	
Luveltamab Tazevibulin (STRO-002)	Sutro Biopharma	1. NCT03748186 (Phase I,136 patients)	Advanced epithelial ovarian cancer and endometrial cancers	SC209 (hemiasterlin)	Tubulin inhibitor	Valine-citrulline	4	Completed. Data awaited	[93]
	Sutro Biopharma	2. NCT05200364 (Phase I, 58 patients)	Advanced epithelial ovarian cancer					On-going	
	Tasly Pharmaceutical Group Co., Ltd	3. NCT06238687 (Phase I/II, 132 patients)	Advanced epithelial ovarian cancer, endometrial cancer and other advanced solid tumors					On-going	
	Sutro Biopharma	4. NCT06679582 (Phase I/II, 24 patients)	Acute myeloid leukemia (AML)					On-going	
	Sutro Biopharma	5. NCT06555263 (Phase II, 43 patients)	Non-small cell lung cancer					On-going	
	Sutro Biopharma	6. REFRaME-O1; NCT05870748 (Phase II/III, 600 patients)	Ovarian cancer expressing FOLR1					On-going	
ZW191	Zymeworks BC Inc	NCT06555744 (Phase I, 145 patients)	Advanced solid tumors	ZD06519	TOP1 inhibitor	GGFG-aminomethyl	8	Ongoing	[94]
BAT8006	Bio-Thera Solutions Ltd	1. NCT05378737 (Phase I, 216 patients)	Advanced solid tumors	NA	TOP1 inhibitor	NA	8	On-going	[95]
	Bio-Thera Solutions Ltd	2. NCT06545617 (Phase I/II, 170 patients)	Platinum-resistant ovarian cancer					On-going	
IMGN151	AbbVie	NCT05527184 (Phase I, 423 patients)	Advanced solid tumors	DM21 (maytansine)	Tubulin inhibitor	NA	3.5	On-going	[96]
AMT-151	Multitude Therapeutics Inc.	NCT05498597 (Phase I, 30 patients)	Selected advanced solid tumors	NA	NA	NA	NA	On-going	NA
LY4170156 (MBK-103)	Eli Lilly (ex Mablink)	NCT06400472 (Phase I, 220 patients)	Selected Advanced solid tumors	Exatecan	TOP1 inhibitor	NA	8	On-going	[97]

*Abbreviations: DAR, drug to antibody ratio, FDA, US Food and Drug Administration;* TOP1: Topoisomerase 1; *NA, not available.*

Reported adverse events have been similar throughout the literature. Ocular toxicities, particularly corneal keratopathy and blurred vision, were the most commonly reported adverse events, and a common side effect of DM4-based therapeutics. The mechanism behind this ocular toxicity remains unclear (and may be unrelated to FRα), but preventive measures such as topical corticosteroid eye drops can reduce the severity of ocular adverse events.^100^

#### MORAb-202

MORAb-202 is an ADC formed by the conjugation of farletuzumab to the tubulin inhibitor eribulin (drug-to-antibody ratio (DAR) of 4) via a cathepsin-cleavable Val-Cit linker^101^ (Figure 4). It is the first ADC therapy to use eribulin as a payload. Eribulin is approved in the USA and Europe for the treatment of metastatic breast cancer for third-line treatment and beyond.^102^ It has a novel mode of action that is distinct from that of other tubulin inhibitors: it terminates microtubule elongation to trigger cell death with no effect on microtubule depolymerization.^103^ MORAb-202 demonstrated efficacy in FRα-positive models across multiple solid tumors, with evidence of bystander killing *in vivo*.^101,104^ A Phase 1 study (NCT03386942) of MORAb-202 demonstrated antitumor activity in multiple tumor types and identified bone marrow toxicity and interstitial lung disease (ILD) as the main adverse events of interest.^89^ Two Phase 2 studies are ongoing to evaluate the efficacy of MORAb-202 in patients with metastatic lung adenocarcinoma (NCT05577715) and PROC (NCT05613088) (Table 2).

#### Luveltamab Tazevibulin

Luveltamab Tazevibulin (STRO-002), is a novel FRα-targeting ADC under clinical investigation for ovarian and endometrial cancers. Using Sutro’s XpressCF+ platform and copper-free click chemistry, STRO-002 was developed by conjugating a novel cleavable 3-aminophenyl hemiasterlin linker warhead (SC239) to para-azidomethyl-L-phenylalanine (pAMF), a nonnatural amino acid incorporated at specific sites within the anti-FRα antibody backbone (SP8166).^105^ This ADC has several notable features. First, the payload SC209, acts as both a potent tubulin inhibitor and as a weaker substrate for the drug resistance-related cellular efflux pump P-gp. This makes SC209 an appealing payload in treatment-resistant disease.^105^ Moreover, the conjugation sites in STRO-002 were selected with careful consideration for antibody stability, efficiency of pAMF incorporation and payload binding, as well as *in vivo* pharmacokinetics. Two sites on the heavy chains (HC-Y180 and HC-F404) were chosen to generate an optimized, homogenous ADC with a DAR of 4 (Figure 4). STRO-002 has been shown to be remarkably stable in the circulation, with no change in DAR for up to 21 days and a half-life of 6.4 days in mice. Single doses of STRO-002 can induce significant growth inhibition in patient-derived xenografts, with its activity enhanced when combined with the chemotherapeutic agent carboplatin or with the anti-VEGF antibody bevacizumab that targets tumor vasculature.^105^ STRO-002 has been evaluated in several trials (e.g., NCT03748186, NCT05200364) and more recently has advanced to late-stage clinical trials (e.g., NCT06555263, NCT05870748).

#### Other ADCs

**Rinatabart sesutecan (PRO1184)** is a human anti-FRα antibody conjugated to the topoisomerase 1 (TOP1) inhibitor exatecan with a novel proprietary hydrophilic linker (DAR 8) (Figure 4). Previous studies have suggested that this hydrophilic linker confers superior physiological properties and pharmacokinetic profiles compared to conventional linkers. PRO1184 exerted potent cytotoxic activity in murine xenograft models across a range of FRα expression levels, consistent with on-target potency as well as bystander activity.^92^ The combined Phase 1/2 PRO1184–001 study (NCT05579366) is currently underway for patients with advanced solid tumors.

**AZD5335** is a FRα-targeting antibody conjugated to the AstraZeneca’s proprietary topoisomerase 1 (TOP1) inhibitor AZ14170132, with a homogeneous DAR of 8 (Figure 4). The TOP1i payload inhibits DNA repair to induce apoptosis. This ADC has shown robust anti-tumor activity in patient-derived xenografts with a range of FRα expression levels.^91^ A Phase 1/2a study of AZD5335 (FONTANA, NCT05797168) is currently recruiting.

**ZW191** is an anti-FRα ADC comprising a novel fully humanized IgG1 antibody covalently conjugated to ZD06519, a new camptothecin-based topoisomerase 1 (TOP1) inhibitor via maleimide-connected tetrapeptide cleavable linker with a DAR of 8. This ADC featured a favorable binding affinity and superior tumor tissue penetration due to its optimized antibody moiety, along with effective bystander killing activity via its payload. Encouraging efficacy data and tolerability profile in non-human primate studies permitted the translation of ZW191 to a Phase 1 clinical trial in participants with advanced malignancies, including ovarian, endometrial, and non-small cell lung cancers (NCT06555744).^94^

**BAT8006** is an additional anti-FRα ADC incorporating topoisomerase 1 (TOP1) inhibitor payloads with a DAR of 8, which has also advanced beyond preclinical studies to a Phase 2 trial, BAT8006, which is underway in PROC, fallopian tube, or primary peritoneal cancers^95^ (Figure 4).

**IMGN151** is a lead ADC developed by ImmunoGene featuring an asymmetric, bivalent, biparatopic antibody. It targets two non-overlapping epitopes of FRα and is conjugated to the highly potent maytansinoid derivative DM21 via a stable cleavable peptide linker, with an average DAR of 3.5 (Figure 4). This ADC has shown enhanced binding and internalization compared to its parent monospecific antibodies. Moreover, it has demonstrated antitumor potency in ovarian cancer xenografts with varied FRα expression levels. A Phase 1 study is in process (NCT05527184).^96^

**MBK-103 (LY4170156)** comprises an Fc-attenuated humanized IgG1 antibody targeting FRα and a novel polysarcosine hydrophilic masking entity, allowing the conjugation of the exatecan payload with a DAR of 8^97^ (Figure 4).

### Combination therapeutic strategies

#### ADC combinations with chemotherapy

The majority of FRα-targeting monoclonal antibodies have been deployed with chemotherapy to maximize efficacy. However, to date, no monoclonal therapy for FRα has demonstrated a survival benefit over standard chemotherapy in a Phase 3 study. In contrast, combination strategies with ADCs remain comparatively less-well explored. MIRV plus carboplatin combination has demonstrated better anti-tumor efficacy in ovarian cancer patient-derived xenografts than standard chemotherapy combinations, such as carboplatin/taxane.^106^ These data have prompted further evaluation for potential synergies with conventional cytotoxic agents in the clinic: two clinical trials are currently evaluating MIRV in combination with chemotherapy (NCT05456685 and NCT04606914) and may determine a future standard of care, even in platinum-sensitive disease. Similar preclinical data in patient-derived xenograft models of FRα-expressing cancer support the use of STRO-002 and carboplatin combinations, further supporting the potential of such combination approached,^105^ although this ADC has yet to be evaluated in clinical combination studies.

#### Combination of anti-FRα antibodies and ADCs with targeted agents and immunotherapy

Bevacizumab, an anti-vascular endothelial growth factor-A monoclonal IgG1 antibody, has progressively entered widespread use for the frontline treatment of advanced ovarian cancer. As its position in the treatment pathway for ovarian cancer has matured, novel combinations with MIRV are now being evaluated in preclinical models and clinical trials. Preliminary *in vivo* data has demonstrated that MIRV/bevacizumab combination can induce substantial tumor regression, including complete responses, in ovarian cancer xenografts.^106^ A subsequent Phase 1b clinical trial (NCT02606305) showed that MIRV/bevacizumab combination therapy is well tolerated in PROC, with lower incidence of myelosuppressive toxicities relative to conventional cytotoxic regimens.^107^ Additionally, MIRV/bevacizumab appeared to have a higher ORR in patients with moderate or high FRα expression than conventional treatment. Based on these results, the ongoing Phase 3 GLORIOSA clinical trial (NCT05445778) is comparing MIRV/bevacizumab to bevacizumab alone as maintenance therapy in patients with high FRα expression, platinum-sensitive ovarian cancer.^108^ The combination of STRO-002 and bevacizumab is also under investigation in a Phase 1, open-label clinical trial in ovarian cancer (NCT05200364).

Immunotherapy combination strategies are also under investigation. While PD-1 inhibitors such as pembrolizumab have shown impressive, sustainable responses in a host of solid tumors, these drugs have so far had considerably more modest benefits in gynecological cancers. However, an ongoing Phase 2 clinical trial is underway to investigate whether MIRV could improve the low response rates to single agent pembrolizumab in recurrent or persistent endometrial cancer.^109^ As of a data cutoff in November 2023, of the 16 patients who received this combination, 6 achieved complete or partial responses. Further data will clarify these results as the trial continues. The mechanistic basis for this potential synergy remains uncertain.

#### Other approaches

In addition to anti-FRα ADCs, FRα vaccines have also been considered to help overcome PD-1/PD-L1 therapy resistance in advanced ovarian cancer. One such Phase 2 clinical trial in patients with recurrent ovarian cancer (NCT02764333) combined TPIV200, a peptide vaccine composed of five immunogenic peptide epitopes of FRα, with durvalumab, a PD-L1 inhibitor antibody.^110^ Although treatment was well tolerated and increased early T cell responses were observed, only one patient demonstrated a partial response, and all patients ultimately progressed. Nevertheless, the overall survival duration was notably longer than expected for such a heavily pretreated patient cohort. These data suggest that vaccine approaches are worthy of further investigation, although their role relative to better-established strategies remains uncertain.

## Conclusion

The recent approval of mirvetuximab soravtansine confirms FRα is a *bona fide* target for the treatment of solid tumors and a prime candidate for antibody-based therapies. Driven by its success, several other FRα-targeted ADCs are now rapidly progressing through clinical evaluation. Many of these newly developed ADCs incorporate innovative and potent payloads with unique mechanisms of action that may target specific features of cancer biology and incorporate cutting-edge design features to enhance therapeutic efficacy. A host of exciting preclinical findings and clinical trial data further suggest that combining these anti-FRα drugs with chemotherapy, targeted or immune therapy, may be highly efficacious, and some of these may emerge as future standards of care. A deeper understanding of the underlying mechanisms driving these synergies will help optimize treatment regimens and identify parameters for identifying the appropriate patient cohorts. Given its expression across a wide range of advanced solid tumors, we expect FRα will play a growing role in the future of oncology, particularly in advanced disease, where there is the highest unmet need.
